# Chemogenetic inhibition of NTS astrocytes normalizes cardiac autonomic control and ameliorate hypertension during chronic intermittent hypoxia

**DOI:** 10.1186/s40659-023-00463-0

**Published:** 2023-11-06

**Authors:** Katherin Pereyra, Alexandra Las Heras, Camilo Toledo, Esteban Díaz-Jara, Rodrigo Iturriaga, Rodrigo Del Rio

**Affiliations:** 1 Laboratory of Cardiorespiratory Control, Facultad de Ciencias Biológicas, Pontificia Universidad Católica de Chile, Santiago, Chile; 2 Instituto de Fisiología, Universidad Austral de Chile, Valdivia, Chile; 3 Centro de Investigación en Fisiología y Medicina en Altura, Facultad de Ciencias de la Salud, Universidad de Antofagasta, Antofagasta, Chile; 4 Centro de Excelencia en Biomedicina de Magallanes (CEBIMA), Universidad de Magallanes, Punta Arenas, Chile; 5 Department of Cell Biology and Physiology, School of Medicine, University of Kansas Medical Center, Kansas City, KS USA

**Keywords:** Obstructive sleep apnea, Intermittent hypoxia, Astrocytes, Nucleus of the solitary tract, Reactive astrocytes, Neuroinflammation

## Abstract

**Background:**

Obstructive sleep apnea (OSA) is characterized by recurrent episodes of chronic intermittent hypoxia (CIH), which has been linked to the development of sympathoexcitation and hypertension. Furthermore, it has been shown that CIH induced inflammation and neuronal hyperactivation in the nucleus of the solitary tract (NTS), a key brainstem region involved in sympathetic and cardiovascular regulation. Since several studies have proposed that NTS astrocytes may mediate neuroinflammation, we aimed to determine the potential contribution of NTS-astrocytes on the pathogenesis of CIH-induced hypertension.

**Results:**

Twenty-one days of CIH induced autonomic imbalance and hypertension in rats. Notably, acute chemogenetic inhibition (CNO) of medullary NTS astrocytes using Designer Receptors Exclusively Activated by Designers Drugs (DREADD) restored normal cardiac variability (LF/HF: 1.1 ± 0.2 vs. 2.4 ± 0.2 vs. 1.4 ± 0.3, Sham vs. CIH vs. CIH + CNO, respectively) and markedly reduced arterial blood pressure in rats exposed to CIH (MABP: 82.7 ± 1.2 vs. 104.8 ± 4.4 vs. 89.6 ± 0.9 mmHg, Sham vs. CIH vs. CIH + CNO, respectively). In addition, the potentiated sympathoexcitation elicit by acute hypoxic chemoreflex activation in rats exposed to CIH was also completely abolished by chemogenetic inhibition of NTS astrocytes using DREADDs.

**Conclusion:**

Our results support a role for NTS astrocytes in the maintenance of heightened sympathetic drive and hypertension during chronic exposure to intermittent hypoxia mimicking OSA.

**Supplementary Information:**

The online version contains supplementary material available at 10.1186/s40659-023-00463-0.

## Background

Obstructive sleep apnea (OSA) is a common sleep disorder that affects approximately 20% of the adult population worldwide [1]. It is characterized by recurrent episodes of partial or complete obstruction of the upper airway during sleep, resulting in chronic intermittent hypoxia (CIH) and sleep fragmentation [2]. OSA has been linked to a range of adverse health outcomes, including cardiovascular disease, neurocognitive impairment, and metabolic disorders [3, 4]. Thus, OSA is complex and involves interactions between various physiological systems, including the respiratory, autonomic, and central nervous systems.

The evidence suggests that CIH, one main pathophysiological mechanism of OSA, enhances peripheral chemoafferent activity to the nucleus of the solitary tract (NTS), an integrative nucleus that communicate with main centers involve in autonomic control that participate in the increase sympathetic outflow and systemic hypertension [3, 5]. Indeed, CIH has been shown to increase the activity of neurons located in the caudal aspect of the NTS, leading to enhanced sympathetic activity, respiratory instability, and high blood pressures [6–15]. The precise mechanism(s) underpinning the effects of OSA on the neural control of hemodynamic function are still not completely understood. However, OSA-induced hypertension has been linked to systemic oxidative stress and inflammation [16–19]. Previously, we reported that increased peripheral chemoreceptor tonic discharges following exposure to CIH induced neuronal activation in the NTS [20]. Later, Oyarce & Iturriaga 2018 provided evidence supporting the presence of neuroinflammation in the NTS of rats exposed to CIH [21]. While the specific nature of NTS neuroinflammation following CIH has never been studied, the role of astrocytes in brain neuroinflammation is widely known [22]. Indeed, recent evidence suggested that astrocytes play a pivotal role in neuroinflammation in neurodegenerative and cardiovascular diseases [23–27]. It has been shown that astrocytes mediates the production of pro-inflammatory cytokines within the central nervous system which in turn modulate the activity of microglia contributing to the generation of a pro-inflammatory niche [28]. Despite the evidence, the possible role of astrocytes in the development of OSA-induced hypertension is not known. Therefore, we aimed to determine for the first time, the potential involvement of the NTS-astrocytes in the pathogenesis of CIH-induced hypertension. Particularly, we aimed to determine if selective chemogenetic inhibition of astrocytes residing within the caudal NTS using Designer Receptors Exclusively Activated by Designer Drugs (DREADD) improves cardiac autonomic control and reduces the hypertension in rats exposed to CIH.

## Results

**Fig. 1:**
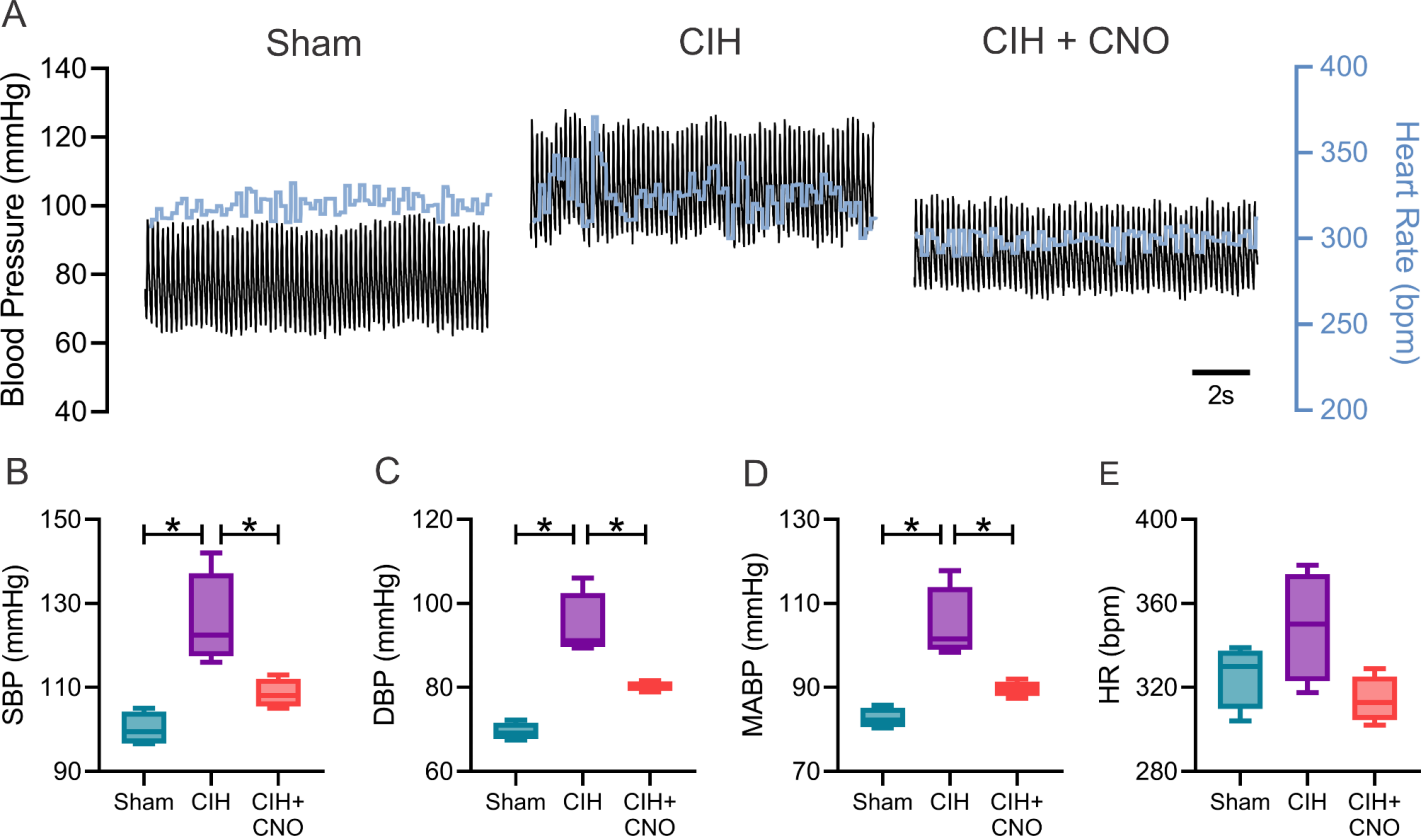
Chemogenetic inhibition of NTS astrocytes abolished hypertension following CIH. **(A)** Representative blood pressure and heart rate recordings at normoxic conditions (F_i_O_2_ 21%). Summary data showing **(B)** systolic (SBP) and **(C)** diastolic blood pressure (DBP), **(D)** mean arterial blood pressure (MABP), **(E)** and heart rate (HR). Data presented as mean ± standard error mean (SEM). *, p < 0.05; One-Way ANOVA for repeated measurements followed by Holm-Sidak *pos hoc* test. N = 4

**Fig. 2:**
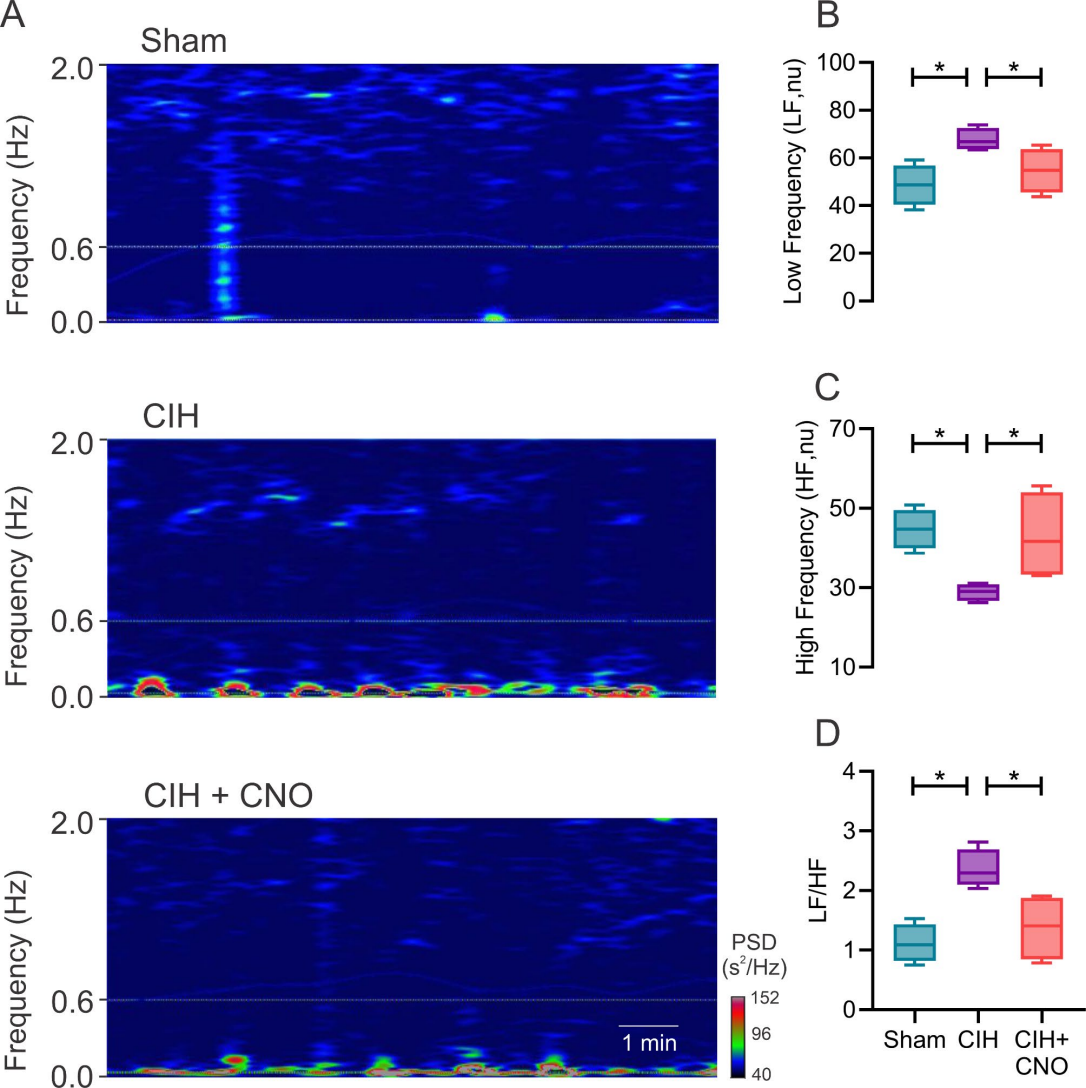
CIH-induced sympatoexcitation is markedly reduced by acute inhibition of NTS astrocytes. **(A)** Representative power spectral density (PSD) spectrum of heart rate variability analysis and quantitative summary data showing cardiac autonomic balance through **(B)** low frequency (LF), **(C)** high frequency (HF) and **(D)** LF/HF ratio spectral components in normoxia (F_i_O_2_ 21%). Data presented as mean ± standard error of the mean (SEM). *, p < 0.05; One-Way ANOVA for repeated measurements followed by Holm-Sidak *pos hoc* test. N = 4

**Fig. 3:**
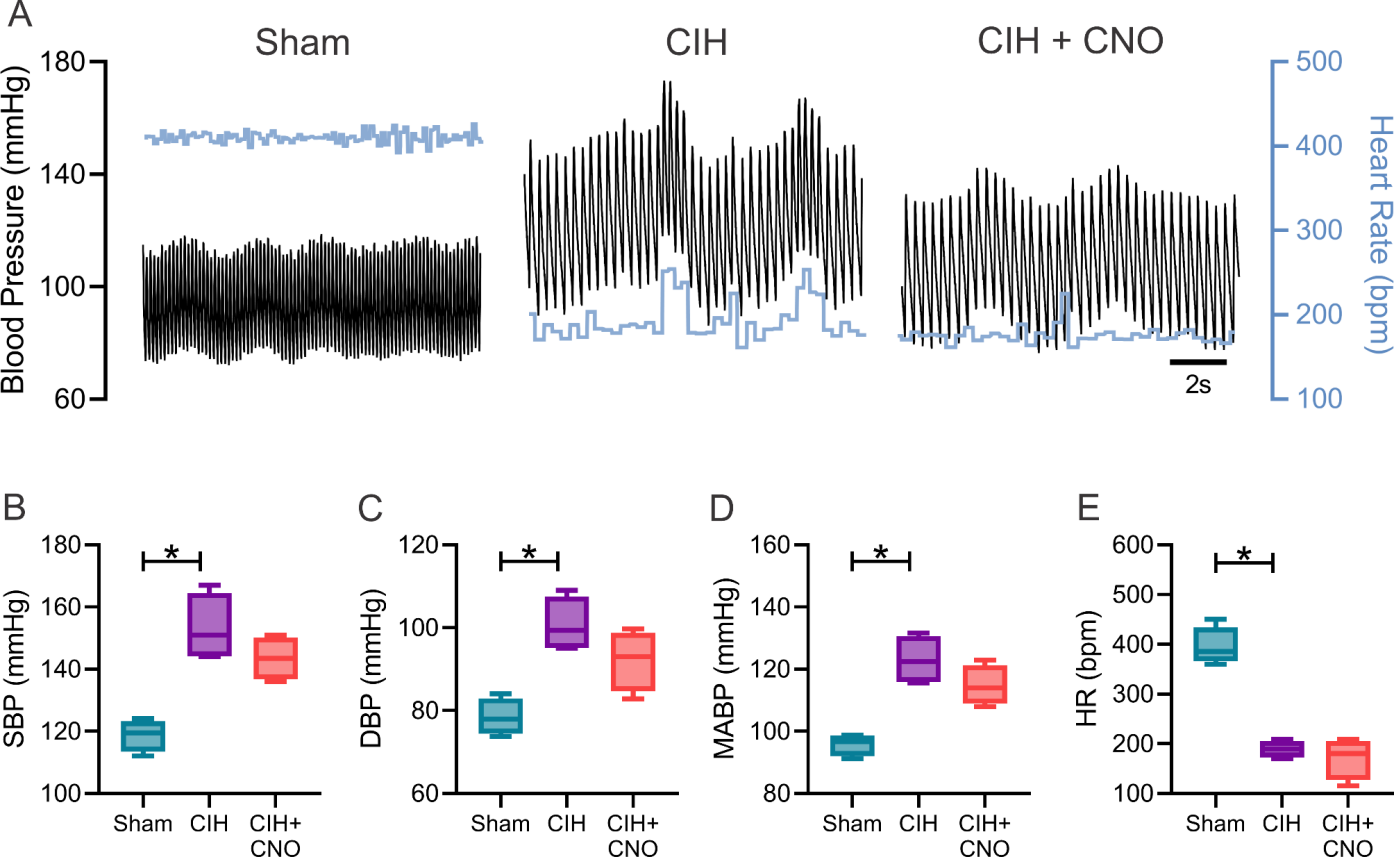
Inhibition of NTS astrocytes blunted the exacerbated vascular response to hypoxia in rats exposed to CIH. **(A)** Representative blood pressure and heart rate recordings during hypoxia (F_i_O_2_ 10%). Summary data showing **(B)** systolic (SBP) and **(C)** diastolic blood pressure (DBP), **(D)** mean arterial blood pressure (MABP), and **(E)** heart rate (HR). Data presented as mean ± standard error of the mean (SEM). *, p < 0.05; One-Way ANOVA for repeated measurements followed by Holm-Sidak *pos hoc* test. N = 4

**Fig. 4:**
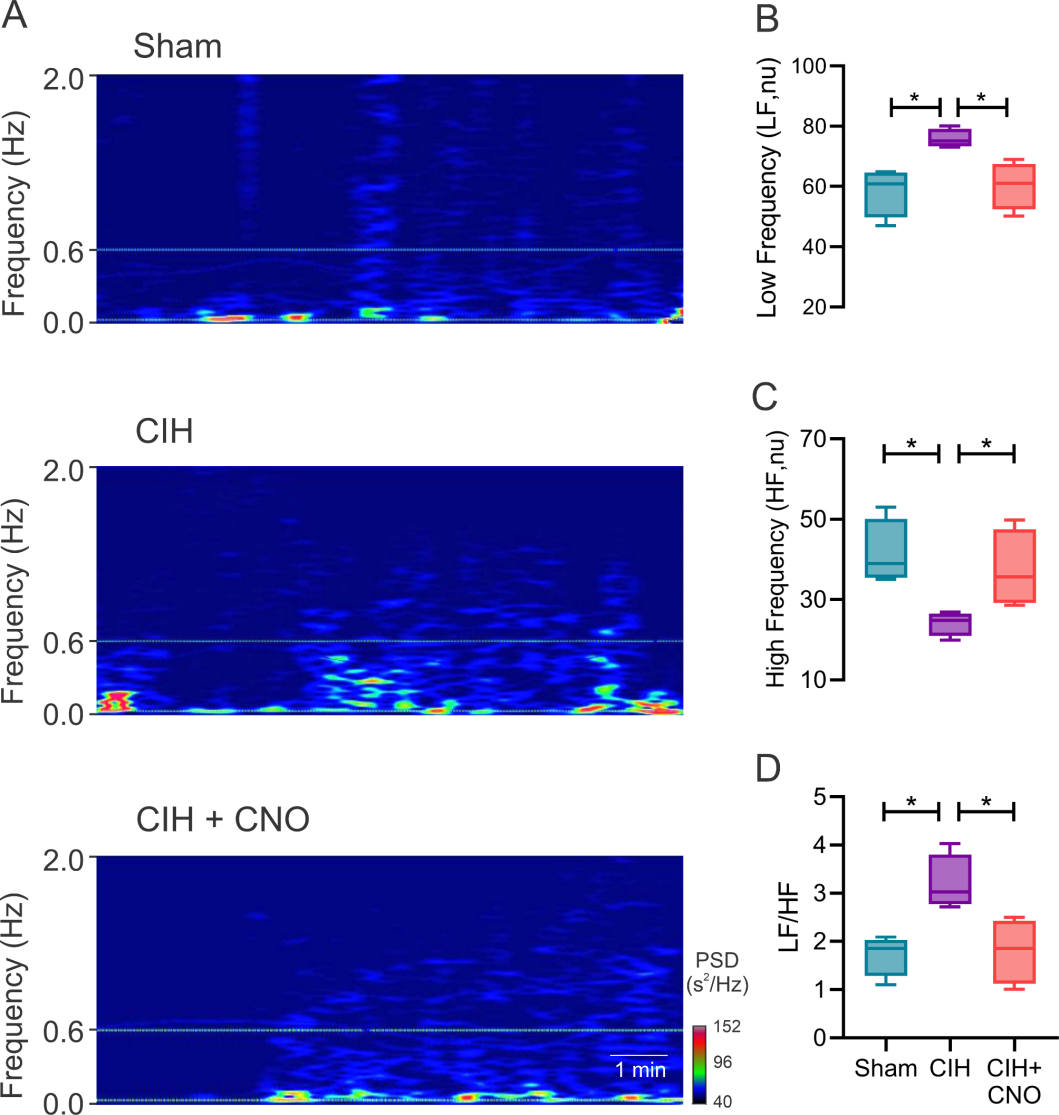
Chemoreflex-induced sympatoexcitation is reduced by chemogenetic inhibition of NTS astrocytes in CIH rats. **(A)** Representative power spectral density (PSD) spectrum of heart rate variability analysis and quantitative summary data showing cardiac autonomic function through **(B)** low frequency (LF), **(C)** high frequency (HF) and (D) LF/HF ratio spectral components in Normoxia (F_i_O_2_ 21%). Data presented as mean ± standard error of the mean (SEM). *, p < 0.05; One-Way ANOVA for repeated measurements followed by Holm-Sidak *pos hoc* test. N = 4

Twenty-one days of CIH results in systemic hypertension (MABP: 82.67 ± 1.21 vs. 104.80 ± 4.39 mmHg, Sham vs. CIH; Fig. 1D) and heart rate variability imbalance (LF/HF: 1.11 ± 0.16 vs. 2.36 ± 0.16, Sham vs. CIH; Fig. 2D) characterized by increases in sympathetic predominance (LF: 48.64 ± 4.2 vs. 67.70 ± 2.37, Sham vs. CIH; Fig. 2A). Acute chemogenetic inhibition of NTS astrocytes following 21 days of CIH results in a significant reduction in MABP (104.80 ± 4.39 vs. 89.63 ± 0.93 mmHg, CIH vs. CIH + CNO; Fig. 1D) and a complete restoration of autonomic balance. Indeed, heart rate variability values of CIH + CNO treated animals were undistinguishable compared to the ones obtained in Sham conditions (LF/HF: 1.11 ± 0.16 vs. 1.38 ± 0.28, Sham vs. CIH + CNO; Fig. 2D). Furthermore, we found that the exacerbated hemodynamic response to hypoxia (F_i_O_2_ 10%) observed in rats exposed to CIH for 21 days (MABP: 95.85 ± 1.81 mmHg vs. 123.00 ± 3.95, Sham vs. CIH; Fig. 3D), was diminished by chemogenetic inhibition of NTS astrocytes (MABP: 123.00 ± 3.95 vs. 114.70 ± 3.20 mmHg, CIH vs. CIH + CNO; Fig. 3D). Hypoxia-induced sympatoexcitation was also found to be enhanced in rats exposed to 21 days of CIH (LF/HF: 1.72 ± 0.21 vs. 3.20 ± 0.29, Sham vs. CIH; Fig. 4D) and NTS astrocyte inhibition using DREADDs abolished the exaggerated sympathetic response to hypoxia (LF/HF: 3.20 ± 0.29 vs. 1.81 ± 0.34, CIH vs. CIH + CNO; Fig. 4D). We also performed experiments in Sham rats that received DREADD injections and CNO as well as in Control rats that only received CNO i.p. injections. We found that activation of DREADDs in Sham rats with CNO (1 mg/kg) has no effects on blood pressure nor in resting heart rate (Supplemental Fig. 1). Furthermore, we found that CNO (1 mg/kg i.p.) has no cardiac effects when injected i.p. in control rats. Indeed, heart systolic and diastolic function remains unaltered following CNO injection (Supplemental Fig. 1 and Supplemental Table 1).

## Discussion

Obstructive sleep apnea (OSA), a prevalent sleep disorder affecting millions of people worldwide, is associated with several cardiovascular and metabolic comorbidities, including hypertension [29–31]. The mechanisms involved in the development/maintenance of OSA-induced hypertension are multifactorial and involves at least, neuroinflammation, oxidative stress, and increased sympathetic drive [31–33]. In the present study, we investigated the role of astrocytes in the NTS, the primary site of integration of cardiovascular information in the brainstem, in the maintenance of high blood pressure during CIH, a main pathophysiological hallmark of OSA.

Our results showed for the first time that selective inhibition of astrocytes residing within the caudal NTS significantly reduced the CIH-induced hypertension in rats. Indeed, animals exposed for 21 days to CIH displayed resting sympathoexcitation and hypertension. Notably, selective inhibition of NTS astrocytes using a chemogenetic approached in freely moving animals results in a significant reduction in blood pressure and a complete restoration of normal heart rate variability in CIH animals. The later strongly supporting a role for NTS astrocytes in the maintenance of both sympathoexcitation and hypertension during CIH. In addition, we found that selective inhibition of NTS astrocytes reduce the exaggerated hemodynamic and sympathetic responses to hypoxia in CIH animals, suggesting that NTS astrocyte may participate in the regulation of the enhanced cardiovascular chemoreflex response observed in CIH. Further experiments are needed to fully determine que contribution of NTS astrocytes on chemoreflex function following CIH.

The precise mechanism by which NTS astrocytes contribute, in part, to the development of hypertension and potentiation of sympathetic drive during CIH remains unclear. However, it has been shown that sustained hypoxia induced NTS plasticity through activation of astrocytes within the NTS [34–36]. Interestingly, it is well-known that CIH increases carotid bodies peripheral chemoreceptor discharges to the NTS, increasing glutamate spillover [20]. Therefore, it is plausible to hypothesize that activation of peripheral chemoreceptors during CIH results in NTS astrocytes reactivity altering synaptic transmission at the level of NTS neurons being the outcome an exaggerated sympathetic drive and systemic hypertension. This interesting hypothesis deserves future investigations.

There are inherent limitations in the present study. We performed acute inhibition of NTS astrocytes in rats exposed to 21 days of CIH. While results showing that acute inhibition of NTS astrocytes offer salutary potential we cannot rule out the effects of chronic astrocyte inhibition on NTS-mediated cardiovascular regulation during CIH. Future studies are needed to fully determine the cardiovascular outcomes after chronic inhibition of NTS astrocytes following CIH. Although out of the scope of the present study, we did not provide any mechanistic insight into the alterations of NTS astrocytes during CIH. Nevertheless, we can speculate that during CIH astrocyte became reactive and promotes a pro-inflammatory niche within the NTS that increases neuronal firing. The later should increase sympathetic discharges being the outcome an increase in blood pressure. Further studies are needed to elucidate the mechanism(s) underlying astrocyte activation and its contribution to sympathoexcitation and hypertension in CIH mimicking OSA.

## Conclusion

Our results showed that chemogenetic inhibition of astrocytes residing within the caudal region of the NTS, restores normal sympathetic drive and significantly reduces hypertension in rats exposed to 21 days of CIH mimicking OSA syndrome. Therefore, targeting medullary astrocytes may offer new avenues for the treatment of high blood pressure and other cardiovascular comorbidities in OSA patients.

## Methods

**Fig. 5:**
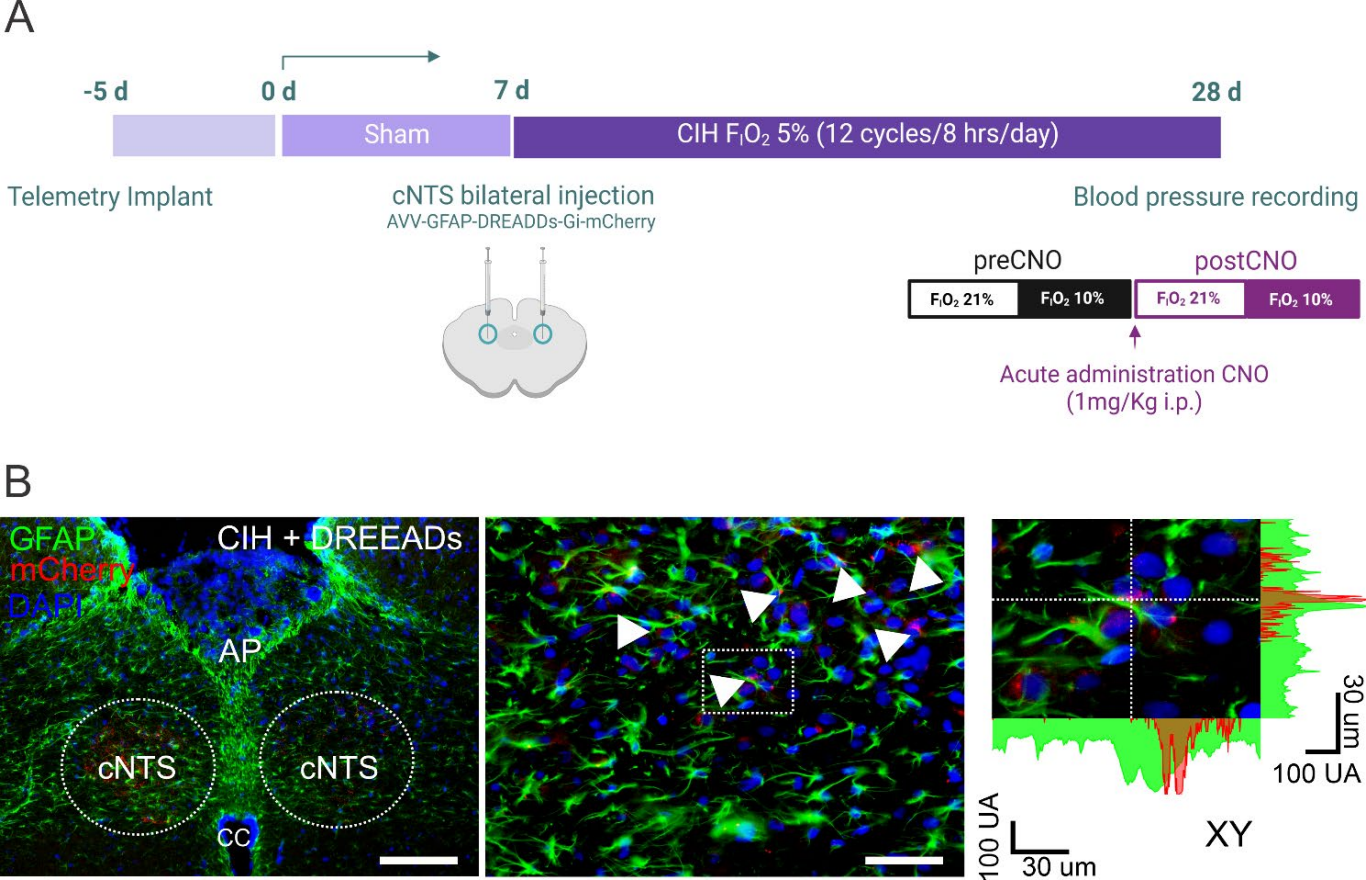
Experimental design. **(A)** Adult male Sprague-Dawley rats were exposed to CIH for 21 days and monitored before CIH and pre/post-acute administration of clozapine N-Oxide (CNO). Animals were injected at 21d post CIH with AVV-GFAP-DREADDs(Gi)-mCherry bilaterally in commissural NTS (cNTS). **(B)** Representative immunostaining showing mCherry, GFAP and DAPI expression (left panel) at cNTS level, scale bar represent 170 μm (left panel) and 30 μm (middle panel). Arrowheads indicate co-localization of astrocytes and DREADDs in 2D and Z-plane. N = 4

Animals: Longitudinal studies were performed in unrestrained, freely moving 4-month-old male Sprague Dawley rats (n = 4) as we show in Fig. 5A. Animals were housed in individual chambers under sham-normoxic conditions first for one week. Then, chambers were flushed for 21 days with intermittent cycles of hypoxia (F_i_O_2_ 5%,12 times/hour, 8 h/day). The O_2_ level inside the chambers was continuously monitored with an oxygen analyzer and the CO_2_ levels and humidity were maintained at low levels by continuous air extraction. At day 21 of CIH, hemodynamic parameters were recorded using indwelling blood pressure radiotelemetry before and after acute inhibition of NTS astrocytes (Fig. 5A). All experimental protocols were approved by the Ethics Committee for Animal Experiments of the Pontificia Universidad Católica de Chile (protocol ID 200,617,015) and were conducted according to National Institutes of Health Guidelines for the Care and Use of Laboratory Animals.

*Stereotaxic surgery and DREADD expression.* Stereotaxic surgery was performed ketamine-xylazine (100 mg/Kg and 10 mg/Kg, respectively) anesthetized animals to delivered bilaterally into the NTS (-14.3 mm to bregma) an adeno-associated virus (450 nL/side, 1*10^12^ vg) containing an inhibitory (Gi) DREADD under the control of the GFAP promoter for selective astrocyte inhibition (Fig. 5B). One-week post-surgery, animals underwent physiological recordings (i.e. blood pressure, heart rate) before and 30 min after a single i.p. injection of clozapine N-oxide (CNO, 1 mg/kg).

*Telemetry and Hypoxic stimulation.* Arterial blood pressure was continuously monitored using radiotelemetry (DSI, USA). Acute cardiovascular response to hypoxia-induced chemoreflex activation was made by allowing the rats to breath hypoxic gas (FiO_2_ 10%) for 10 min at rest.

*Immunofluorescence and DREADDs expression.* 4% paraformaldehyde fixed brainstem tissue was used for inmunohistochemical procedures in free-floating sections (25 μm) containing the NTS. Briefly, sections were first incubated in a PBS-buffered solution with 1% BSA, 0,5% Triton-X, and 2% gelatin from cold water fish skin followed by overnight primary anti-GFAP antibody incubation (rabbit anti-GFAP, 1:1000) at 4 °C. Then, a goat anti-rabbit Alexa 488 (1:1000) antibody was used to visualized GFAP under a confocal microscope.

## Data Analysis

Data is presented as mean ± standard error of the mean (SEM) in the main text. Figures are presented as min to max box plots. Paired One-Way ANOVA was used to evaluate differences between longitudinal data following by Holm-Sidak *post hoc* test in GraphPad Prism 8.0 software (GraphPad Software Inc., La Jolla, CA, USA).

### Electronic supplementary material

Below is the link to the electronic supplementary material.

Supplementary Material 1

## Abbreviations

CIH: Chronic intermittent hypoxia
OSA: Obstructive sleep apnea
MABP: Mean arterial blood pressure
SBP: Systolic blood pressure
DP: Diastolic blood pressure
HR: Heart rate
LF: Low frequency
HF: High frequency
NTS: Nucleus of the solitary tract
DREADDs: Designer receptor exclusively activated by designer drugs
PBS: Phosphate buffer saline
GFAP: Glial fibrillar astrocyte protein
CNO: Clozapine N-oxide
BSA: Bovine serum albumin
