# Supplementary material for: Chemogenetic inhibition of NTS astrocytes normalizes cardiac autonomic control and ameliorate hypertension during chronic intermittent hypoxia
Source: Biol Res. 2023 Nov 6;56:57. doi: 10.1186/s40659-023-00463-0 (PMC10626729; doi:10.1186/s40659-023-00463-0)
Supplement: Supplementary file 1 — Supplementary Material 1 [file 40659_2023_463_MOESM1_ESM.docx]

**Supplemental Material**

**Chemogenetic inhibition of NTS astrocytes normalizes cardiac autonomic control and ameliorate hypertension during chronic intermittent hypoxia**

Katherin Pereyra^1^, Alexandra Las Heras^1^, Camilo Toledo^1,2^, Esteban Diaz-Jara^1^, Rodrigo Iturriaga^1,3^, Rodrigo Del Rio^1,4,5^

^1^Laboratory of Cardiorespiratory Control, Pontificia Universidad Católica de Chile, Santiago, Chile.

^2^Department of Physiology, Universidad Austral de Chile, Valdivia, Chile.

^3^Centro de Investigación en Fisiología y Medicina en Altura. Facultad de Ciencias de la Salud. Universidad de Antofagasta, Antofagasta, Chile.

^4^Centro de Excelencia en Biomedicina de Magallanes (CEBIMA), Universidad de Magallanes, Punta Arenas, Chile.

^5^Department of Cell Biology and Physiology, School of Medicine, University of Kansas Medical Center, Kansas City, Kansas, United States.

**Short title:** Inhibition of NTS astrocytes abolished hypertension in intermittent hypoxia induced OSA-like model.

Corresponding author: Rodrigo Del Rio, Ph. D.

Laboratory of Cardiorespiratory Control

Facultad de Ciencias Biológicas

Pontificia Universidad Católica de Chile.

Santiago, Chile.

Phone: +562 23542859

E-mail: [rdelrio@bio.puc.cl](mailto:rdelrio@bio.puc.cl)

**Supplemental Figure 1**


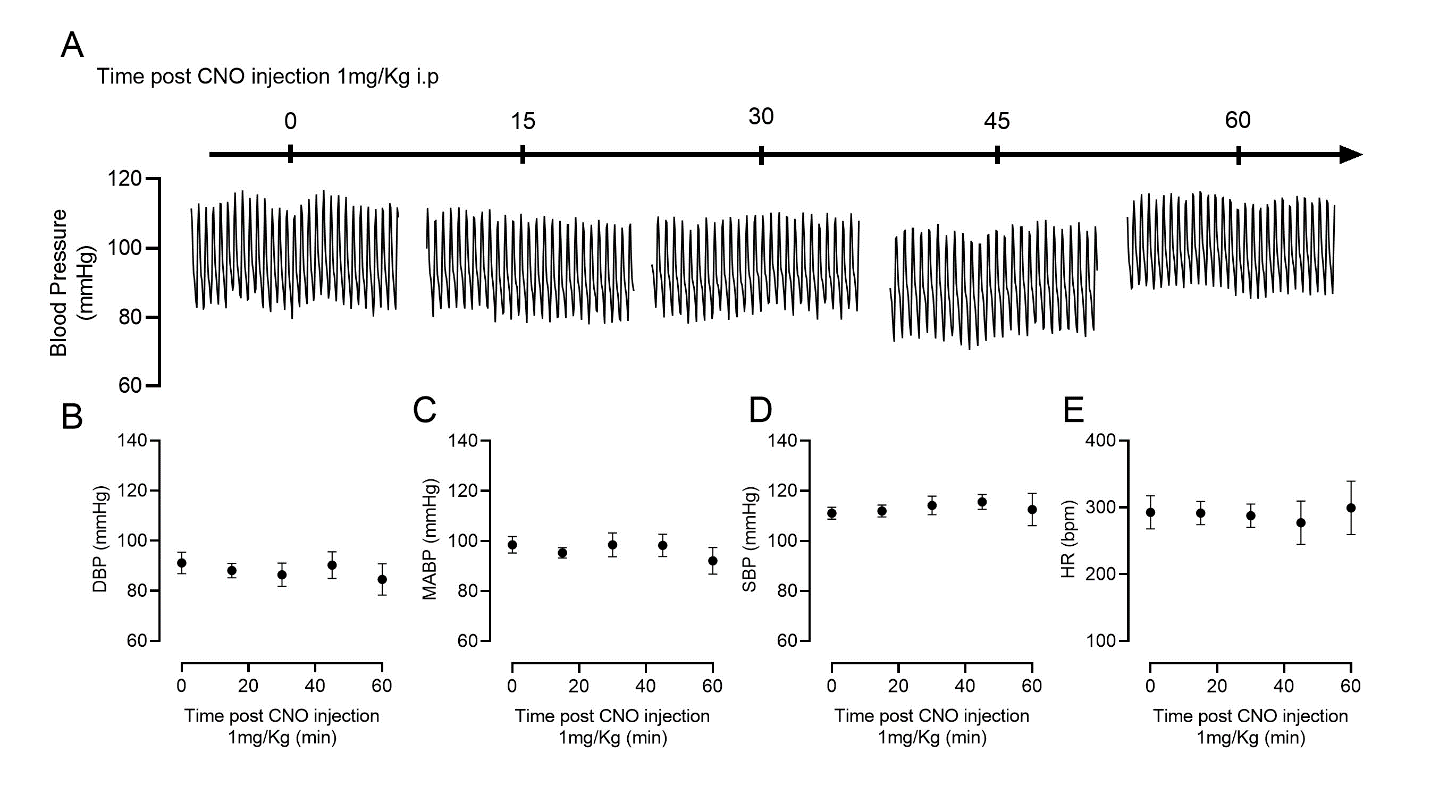


Figure 1. Cardiovascular effects of intraperitoneal CNO administration. (A) Representative blood pressure and heart rate recording from one animal, 1 hour extension. Summary data of (B) diastolic (DBP), (C) systolic (SBP), (D) mean arterial (MABP) blood pressure and (E) heart rate (HR). One-Way ANOVA for repeated measurements, non-significant differences. N=4.


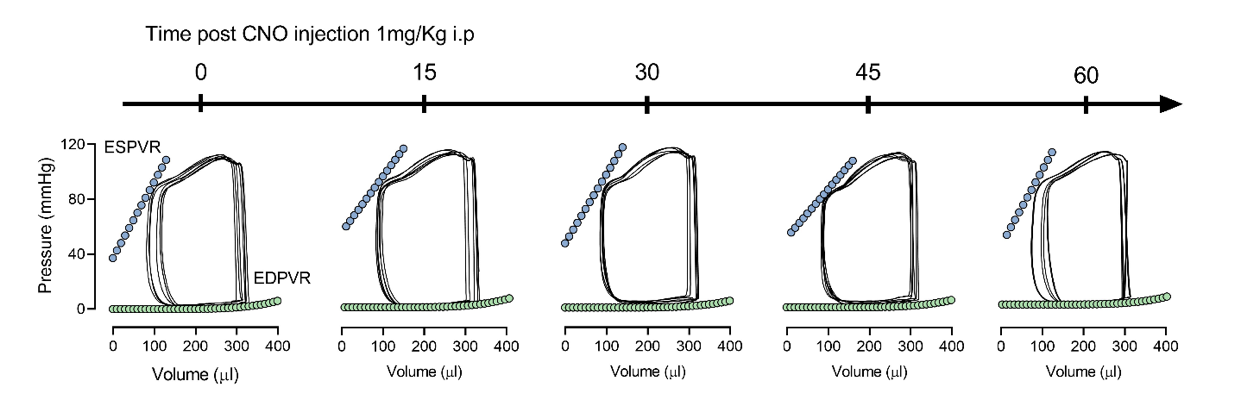
**Supplemental Figure 2**

Figure 2. Cardiac effects after acute i.p. injection of Clozapine N-oxide. In this figure we present representative pressure-volume loops from one animal exposed to CNO and their respective end systolic pressure volume relationship (blue light) and end diastolic pressure volume relationship (green light).

Supplemental Table 1

| Time post CNO injection (1mg/Kg i.p.) | 0 min  (n=4) | 15 min  (n=4) | 30 min  (n=4) | 45 min  (n=4) | 60 min  (n=4) |
| --- | --- | --- | --- | --- | --- |
| CO (ml/min) | 7.8 ± 7.5 | 7.4 ± 6.4 | 7.6 ± 5.5 | 8.4 ± 13.3 | 7.8 ± 9.3 |
| SV (µL) | 297.9 ± 34.6 | 292.8 ± 35.3 | 299.1 ± 33.2 | 297.2 ± 37.2 | 280.3 ± 27.9 |
| EDV (µL) | 393.1 ± 31.1 | 409.1 ± 28.8 | 414.8 ± 28.7 | 383.2 ± 33.5 | 387.3 ± 26.1 |
| ESV (µL) | 95.3 ± 5.2 | 116.4 ± 31.1 | 115.7 ± 26.9 | 92.7 ± 6.7 | 107.0 ± 24.1 |
| ESP (mmHg) | 96.6 ± 3.0 | 95.9 ± 3.9 | 95.4 ± 4.2 | 97.1 ± 3.2 | 97.9 ± 3.9 |
| EDP (mmHg) | 3.9 ± 0.5 | 3.8 ± 0.4 | 3.9 ± 0.3 | 3.8 ± 0.6 | 4.1 ± 0.8 |
| HR (bpm) | 266.4 ± 23.3 | 256.8 ± 16.9 | 260.0 ± 18.9 | 281.7 ± 26.7 | 261.8 ± 17.3 |
| EF (%) | 83.9 ± 4.7 | 79.3 ± 8.9 | 77.9 ± 7.9 | 83.6 ± 10.7 | 76.8 ± 11.2 |
| Ea (mmHg/ µL) | 0.26 ± 0.03 | 0.27 ± 0.03 | 0.25 ± 0.03 | 0.26 ± 0.02 | 0.29 ± 0.03 |
| dP/dt max (mmHg/s) | 8428 ± 533 | 8729 ± 967 | 8733 ± 1014 | 9436 ± 564 | 9303 ± 893 |
| dP/dt min (mmHg/s) | -5491 ± 553 | -5074 ± 476 | -4894 ± 440 | -5318 ± 205 | -5381 ± 151 |
| dV/dt max (µL/s) | 9820 ± 1292 | 9422 ± 544 | 9086 ± 742 | 9633 ± 1214 | 9916 ± 1172 |
| dV/dt min (µL/s) | -10931 ± 1572 | -11069 ± 2444 | -11269 ± 1486 | -10220 ± 1486 | -10311 ±1663 |
| ESPVR | 1.0 ± 0.03 | 0.9 ± 0.2 | 0.9 ± 0.2 | 1.2 ± 0.1 | 1.1 ± 0.2 |
| V0 (µL) | 0.21 ± 0.02 | 0.22 ± 0.02 | 0.22 ± 0.02 | 0.22 ± 0.01 | 0.21 ± 0.01 |

Data is presented as mean ± SEM. CO, cardiac output. SV, stroke volume. EDV, end diastolic volume. ESV, end systolic volume. ESP, end systolic pressure. EDP, end diastolic pressure. HR, heart rate. EF, ejection fraction. Ea, elastance. ESPVR, end systolic pressure volume relationship. One-Way ANOVA for repeated measurements.
